# Supplementary material for: Functional Link between miR-200a and ELK3 Regulates the Metastatic Nature of Breast Cancer
Source: Cancers (Basel). 2020 May 13;12(5):1225. doi: 10.3390/cancers12051225 (PMC7281469; doi:10.3390/cancers12051225)
Supplement: Supplementary file 1 [file cancers-12-01225-s001.pdf]

A Functional Link Between miR-200a and ELK3 Regulates the Metastatic Nature of Breast Cancer

Hyung-Keun Kim, Joo Dong Park, Seung Hee Choi, Dong Jun Shin, Sohyun Hwang, Hae-Yun Jung and Kyung-Soon Par k

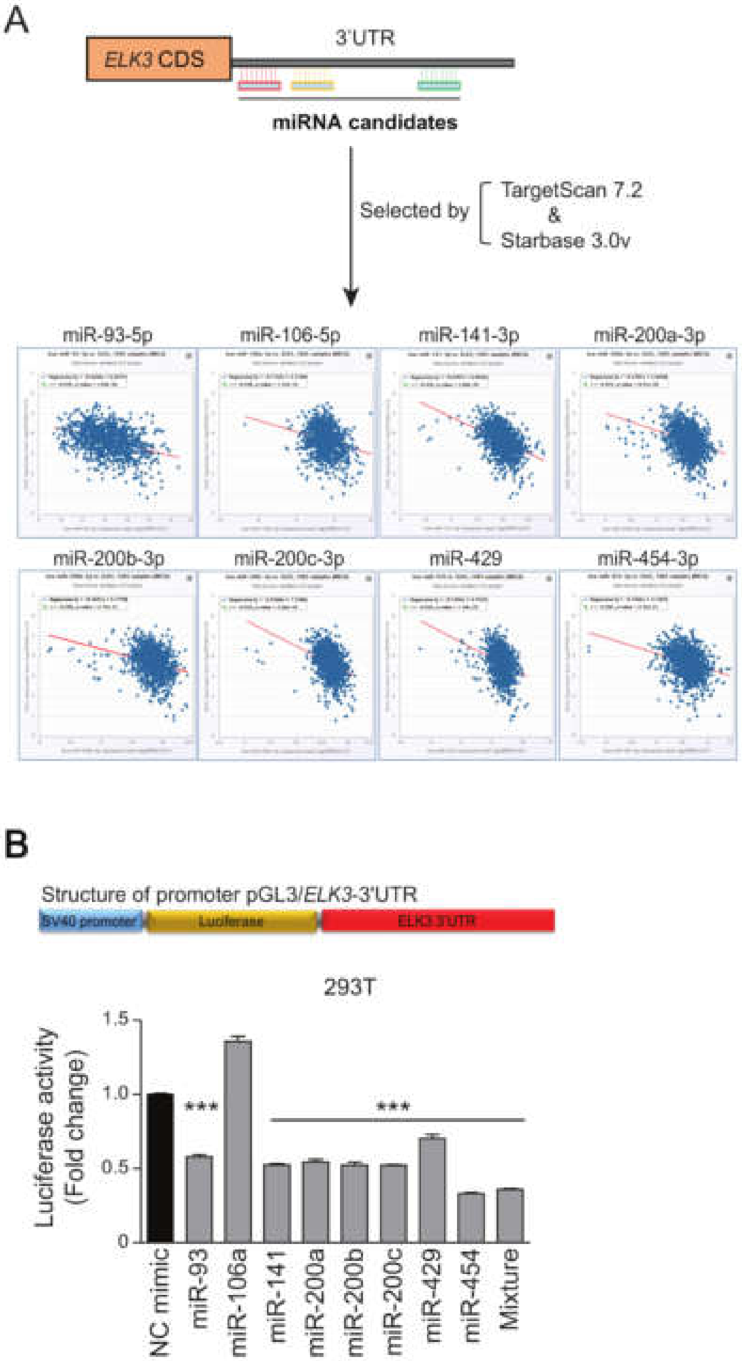

Figure S1. Candidate miRNAs that regulate the *ELK3* mRNA level identified by bioinformatics analyses. (a) Screening of candidate miRNAs was performed using the TargetScan v7.2 and starBase v3.0 software packages. TargetScan predicted several candidate miRNAs. The starBase database was used to select candidate miRNAs with expression levels that were negatively associated with those of the *ELK3* mRNA. Eight miRNAs (miR-93-5p, miR-106-5p, miR-141-3p, miR-200a-3p, miR-200b-3p, miR-200c-3p, miR-429, and miR-454-3p) were selected for further analysis. (b) The candidate miRNAs were co-transfected into 293T cells with a pGL3/ELK3-3′UTR reporter plasmid, and luciferase activity was analyzed after transfection for 48 h. Error bars represent the S.E.M.; ***P < 0.001 (Student's t-test).

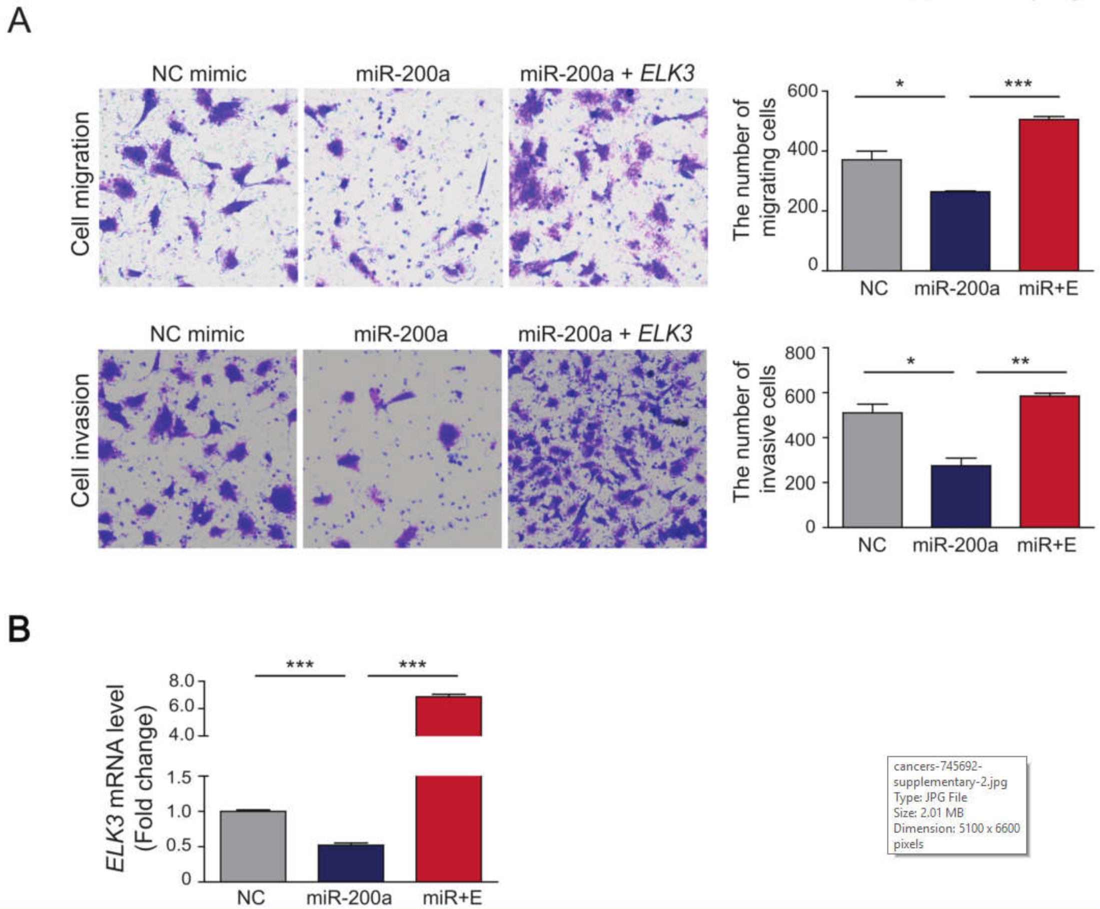

Figure S2. The miR-200a/*ELK3* axis regulates the invasion and migration in Hs578T cells. (a) Representative images showing the cell migration and invasion of Hs578T cells expressing a negative control (NC) mimic, miR-200a (100 nM), or miR-200a (100 nM) plus pcDNA3.1-Flag-*ELK3* (30 ng). The graph indicates quantified cell migration and invasion. Migrating and invasive cells were counted in four randomly selected regions. Scale bars, 20μm. Error bars represent the S.E.M.; *P < 0.05, **P < 0.01 and ***P < 0.001. (b) Quantitative analyses of *ELK3* mRNA levels following the indicated treatments. Error bars represent the S.E.M.; ***P < 0.001 (Student's t-test).

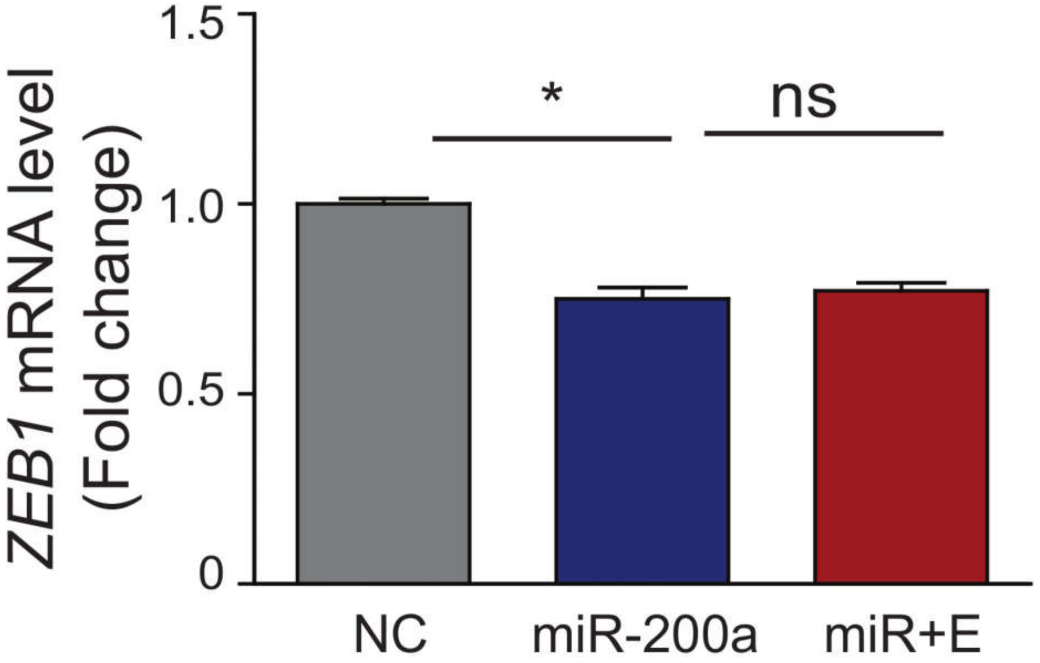

Figure S3. The miR-200a/*ELK3* axis regulates *ZEB1* mRNA level. Quantitative analyses of *ZEB1* mRNA levels following the indicated treatments. Error bars represent the S.E.M.; *P < 0.05 (Student's t-test).
